# Adherence to the Mediterranean Diet and the Risk of Head and Neck Cancer: A Systematic Review and Meta-Analysis of Case–Control Studies

**DOI:** 10.3390/nu17020287

**Published:** 2025-01-14

**Authors:** Nader Zalaquett, Irene Lidoriki, Maria Lampou, Jad Saab, Kishor Hadkhale, Costas Christophi, Stefanos N. Kales

**Affiliations:** 1 Faculty of Medicine, American University of Beirut, Beirut 1107, Lebanon; ngz04@mail.aub.edu (N.Z.); jts09@mail.aub.edu (J.S.); 2 Department of Environmental Health, Harvard T.H. Chan School of Public Health, Boston, MA 02115, USA; khadkhale@hsph.harvard.edu (K.H.); costas.christophi@cut.ac.cy (C.C.); skales@hsph.harvard.edu (S.N.K.); 3 Department of Occupational Medicine, Cambridge Health Alliance, Cambridge, MA 02145, USA; 4 Division of Infectious Diseases, Massachusetts General Hospital, Boston, MA 02114, USA; mlampou@mgh.harvard.edu; 5 Division of Infectious Diseases, Harvard Medical School, Boston, MA 02115, USA; 6 Unit of Health Sciences (Epidemiology), Faculty of Social Sciences, Tampere University, 33520 Tampere, Finland; 7 Cyprus International Institute for Environmental and Public Health, Cyprus University of Technology, 3041 Limassol, Cyprus

**Keywords:** Mediterranean diet, head and neck cancer, oral cancer, dietary pattern, adherence, meta-analysis

## Abstract

Background/Objectives: Head and neck cancer (HNC) is the seventh most common cancer worldwide, with rising incidence rates and significant mortality. While tobacco use, alcohol consumption, and viral infections are established risk factors, the role of dietary patterns, particularly adherence to the Mediterranean diet (MD), in HNC prevention has gained increasing attention. The aim of the current systematic review and meta-analysis is to investigate the association between adherence to the MD and the risk of HNC. Methods: A comprehensive search was conducted, following PRISMA guidelines, to identify relevant studies published up to January 2024 that assessed the association between MD adherence and HNC risk in adults. Pooled odds ratios (OR) for a three-unit increase in MD adherence scores and corresponding 95% confidence intervals (CI) were calculated using a random-effects model. Study quality was assessed using the Newcastle–Ottawa Scale (NOS). Results: Eleven case–control studies comprising 6106 HNC cases and 9166 controls met the inclusion criteria for the systematic review. High adherence to the MD was significantly associated with a reduced risk of HNC (pooled OR = 0.561, 95% CI: 0.368–0.856, *p* = 0.007, I^2^ = 92%). Individual component analyses from three studies revealed that higher fruit and vegetable consumption significantly decreased HNC risk, whereas legumes, fish, and low meat intake showed no statistically significant associations. Conclusions: Adherence to the Mediterranean diet is associated with a significantly reduced risk of head and neck cancer. These findings support the role of the MD in cancer prevention and highlight the potential benefits of MD adherence in reducing HNC risk. Further prospective studies and randomized controlled trials are needed to confirm these findings and explore the underlying mechanisms.

## 1. Introduction

Head and neck cancer (HNC) ranks as the seventh most prevalent type of cancer worldwide, with over 660,000 new cases and 325,000 fatalities each year. The incidence of HNC is steadily increasing and is projected to rise by 30% by 2030 [1,2]. HNC involves malignancies of the oral cavity, pharynx, and larynx, and its rise in global incidence has led to substantial research into modifiable risk factors that would reduce disease occurrence. Well-established modifiable risk factors include tobacco use, alcohol consumption, and viral infections, such as HPV and EBV, collectively accounting for a considerable proportion of HNC cases globally [3,4]. For instance, tobacco smoking and alcohol consumption, especially when combined, are major contributors that are responsible for 72% of cases [3].

Recent epidemiological studies have increasingly highlighted the role of dietary factors in the etiology of HNC. A comprehensive assessment of diet quality is a commonly used approach to understanding the dietary impact by considering interactions among various dietary components, potentially capturing synergistic effects that may predict health outcomes more accurately. For example, plant-based diets rich in fruits, vegetables, and lean proteins have been associated with a reduced risk of developing HNC [5,6,7].

Among various plant-predominant dietary patterns, the Mediterranean diet (MD) has garnered considerable attention for its potential protective effects against the development of chronic diseases, such as diabetes, cardiovascular disease, and cancer [8,9,10,11]. The MD is characterized by a high consumption of fruits, vegetables, legumes, whole grains, nuts, and olive oil; a moderate intake of fish, dairy products, and alcohol, predominantly red wine; and a low intake of red meat and refined foods/sweets. Adherence to the MD can be evaluated utilizing diet quality indexes, such as the Mediterranean Diet Score (MDS), the Alternate Mediterranean Diet Score (aMED), and the Mediterranean Diet Adherence Screener (MEDAS). Studies employing these indexes have linked adherence to the MD with reduced risks of HNC incidence and mortality [12].

The Mediterranean dietary pattern promotes the increased consumption of foods and nutrients that are considered beneficial and contain high levels of polyphenols, carotenoids, vitamins, folates, and flavonoids while discouraging high intake of those deemed unhealthy, such as red and processed meats, sweets, and saturated fat. The proposed mechanisms underlying the health benefits of the MD are mainly based on its anti-inflammatory and antioxidant properties and include the direct inhibition of cancer cells, activation of cell anticancer mechanisms, mitigation of oxidative stress, and reduction in exposure to potentially carcinogenic substances [13,14,15,16,17].

To the best of our knowledge, no prior meta-analysis has been conducted exclusively to evaluate the association between MD adherence and the risk of HNC. Therefore, this meta-analysis aims to systematically review and analyze the current literature on the association between MD adherence and HNC risk.

## 2. Materials and Methods

The Preferred Reporting Items for Systematic Reviews and Meta-Analyses (PRISMA) recommendations were utilized for the design of our meta-analysis (Appendix 1) [18]. Additionally, the Meta-Analyses of Observational Studies in Epidemiology (MOOSE) checklist was applied to ensure methodological rigor (Appendix 2) [19].

### 2.1. Search Strategy

The Medline, Scopus, ClinicalTrials.gov, EMBASE, Cochrane Central Register, and Google Scholar databases were searched exclusively for relevant publications in English up to 10 January 2024. In addition, the chance of including all available articles that met the inclusion criteria was maximized by searching the references of the articles that were retrieved in full text. The main search algorithm that was applied was (“Mediterranean Diet” OR “Olive oil-based diet” OR “Cretan diet” OR “Greek diet” OR “Mediterranean-style eating” OR “Greek cuisine” OR “Cretan cuisine” OR “olive oil”) and (“Head and Neck Cancer” OR “oral cancer” OR “nasal cancer” OR “nasopharyngeal cancer” OR “oropharyngeal cancer” OR “salivary gland cancer” OR “laryngeal cancer” OR “HPV head and neck cancer” OR “squamous cell carcinoma of the head and neck” OR “hypopharyngeal cancer” OR “oral cavity cancer” OR “Craniofacial cancer” OR “Upper aerodigestive tract cancer” OR “Head cancer” OR “throat cancer” OR “maxillofacial cancer” OR “orofacial cancer” OR “paranasal sinus cancer” OR “mandibular cancer” OR “nasal cavity cancer” OR “tonsil cancer”).

The authors predefined the eligibility criteria, and no data restriction was considered during the search procedure. For the systematic review, studies were eligible for inclusion if they met the following criteria: (a) case–control studies in adult patients (over 18 years of age), (b) studies that included patients with head and neck malignancies, and (c) studies that investigated the association between adherence to the Mediterranean dietary pattern and head and neck cancer risk, incidence, and/or mortality. For the meta-analysis, the same inclusion criteria were applied, but only studies published within the last 15 years were included. This time limit was applied due to changes in smoking trends [20], dietary habits [21], and the prevalence of obesity [22] observed over the last 15 years, which could potentially interfere with the results of the meta-analysis. Case reports, experimental animal studies, and reviews were excluded from the present study.

Study search and data tabulation were conducted by three authors on similar predefined forms (N.Z., M.L. and J.S.). Any disagreements between authors were resolved by a fourth and fifth reviewer (I.L. and K.H.). Study selection was performed in three stages. First, duplicate publications were removed, and then the authors read the titles and abstracts of all electronic articles that appeared in the search to assess their eligibility. Then, the authors downloaded the full texts of all articles that met the inclusion criteria, and all case–control studies were selected.

### 2.2. Quality Assessment

The methodological quality of the included articles was assessed independently by two reviewers (I.L and K.H) using the Newcastle–Ottawa Scale (NOS) for case–control studies [23]. This scale is designed to evaluate studies based on three broad categories: the selection of cases and controls, comparability of the groups, and ascertainment of exposure of interest. The selection category examines how cases and controls are chosen and whether they are representative of the population. Comparability assesses whether studies have adequately controlled for confounding factors through methods like matching and/or statistical adjustments. The ascertainment of exposure evaluates the reliability of the exposure measurement and whether it was assessed consistently between cases and controls. Each study is graded using a star system; a higher number of stars indicates higher methodological quality and a lower risk of bias. The possible total points are four points for Selection, two points for Comparability, and three points for Outcomes, with a maximum of nine stars awarded to a study that meets all quality criteria.

### 2.3. Statistical Analysis

The analysis was performed using the Comprehensive Meta-Analysis software version 4 [24]. For consistency, individual study results were extracted and converted to a 3-unit increase in the diet adherence score. The Q-test for heterogeneity was employed to test whether the true effect size is the same in all studies and the I^2^ statistic was also calculated to estimate study heterogeneity. A rough guide to interpretation in the context of meta-analyses of randomized trials is as follows: 0% to 40% might not be important; 30% to 60% may represent moderate heterogeneity; 50% to 90% may represent substantial heterogeneity; and 75% to 100% represent considerable heterogeneity [25]. The alpha level for the Q-test for heterogeneity was set to 0.100 (i.e., a *p*-value < 0.100 was considered statistically significant, indicating that the true effect is different in the studies considered). Due to the increased heterogeneity of the included studies, a random-effects model (DerSimonian‒Laird) [26] using arcsine square root (Freeman–Tukey) transformation was utilized to derive pooled odds ratios (ORs), as well as the corresponding 95% confidence intervals (95% CI). As a sensitivity analysis, results are presented with all studies included, and then, additionally, excluding Samoli et al., an individual component analysis was performed for fruit, vegetables, legumes, fish, and meat consumption. All statistical tests were two-tailed, and significance was set at an alpha level of 0.05.

## 3. Results

### 3.1. Included Studies

**Figure 1:**
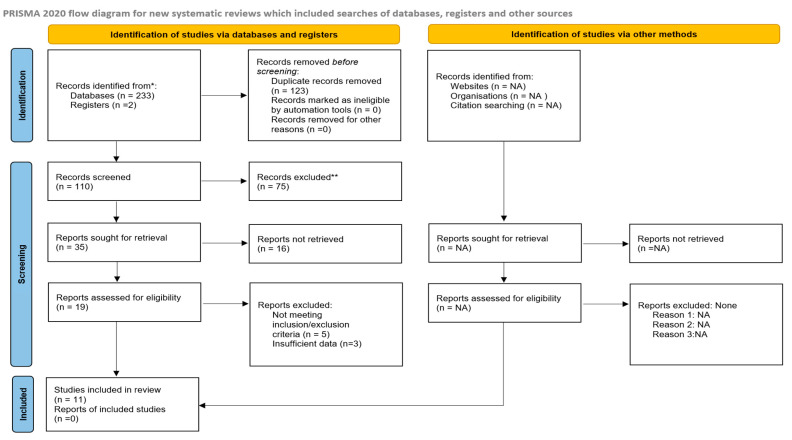
PRISMA flow diagram. * Consider, if feasible to do so, reporting the number of records identified from each database or register searched (rather than the total number across all databases/registers). ** If automation tools were used, indicate how many records were excluded by a human and how many were excluded by automation tools. NA means not applicable.

This systematic review included eleven case–control studies with a total of 6106 head and neck cancer (HNC) cases and 9166 controls [27,28,29,30,31,32,33,34,35,36,37]. The PRISMA flow diagram demonstrates the article selection process (Figure 1). Eight studies were conducted in Europe (Greece, Italy, Switzerland, and Spain) [27,29,30,32,34,35,36,37], two in the United States [33,34], and one in China [31]. The study by Saraiya et al. [28] and Crosignani et al. [36] were excluded from the analysis due to their focus on HNC mortality as the outcome. Moreover, the study by Bosetti et al. [37] was also excluded from the meta-analysis because it was published before the time limit set by the authors for this study (i.e., the last 15 years). The meta-analysis was performed both with and without the study by Samoli et al. [27], which included esophageal cancer patients in addition to HNC patients. Finally, a total of 3405 cases and 5209 controls from seven studies were included in the meta-analysis. All studies assessed adherence to the Mediterranean diet using validated questionnaires.

### 3.2. Quality Evaluation

Eight studies [27,28,29,30,34,35,36] were awarded seven stars and were considered high-quality based on the Newcastle–Ottawa scale, whereas two studies were awarded six stars [31,32], and one study was awarded five stars [33] (Supplementary Table S1).

### 3.3. Data Tabulation

Table 1 presents the characteristics of the included studies, such as the type of study, the number of participants enrolled, and the country where the study took place, as well as the characteristics of the enrolled participants, such as the age and type of cancer. The inclusion criteria and the type of adjustment applied in each study are presented in (Supplementary Table S2).

### 3.4. Mediterranean Diet Adherence Evaluation

Several tools assess adherence to the Mediterranean diet (MD). The Mediterranean Diet Score (MDS) was initially developed to summarize eight characteristics of the traditional Mediterranean diet [40] and was later revised to include fish intake [41]. The MDS and its modified version, the Alternate Mediterranean Diet Score (aMED) [42], employ a nine-component system that emphasizes core MD foods while moderating non-Mediterranean elements. The aMED is specifically adapted for use in non-Mediterranean populations. The Mediterranean Diet Adherence Screener (MEDAS), a 14-item questionnaire, evaluates food frequency and quantities, particularly olive oil, nuts, and fish [43]. The Mediterranean dietary pattern (MDP) evaluates overall dietary patterns, balancing Mediterranean and non-Mediterranean food consumption [44]. Finally, the Mediterranean Adequacy Index (MAI) calculates the ratio of energy from Mediterranean to non-Mediterranean foods, with higher scores reflecting greater adherence [45].

One study evaluated adherence to the Mediterranean diet using the eight-point MDS [37], while five studies used the nine-point MDS [27,28,30,33,34]. One study utilized the aMED score [31], another employed MEDAS [29], and one used a questionnaire adapted from the Spanish Society of Atherosclerosis [32]. Additionally, one study applied three different scores—MDS, MDP, and MAI [35]. Finally, Crosignani et al. [36] used a non-standardized score based on olive oil and butter consumption, combining these variables into an overall score calculated as the tertile of olive oil minus the tertile of butter. The harmonization methods for the different Mediterranean Diet Scores can be found in Supplementary Table S3.

### 3.5. Adherence to the Mediterranean Diet and Head and Neck Cancer Risk

**Figure 2:**
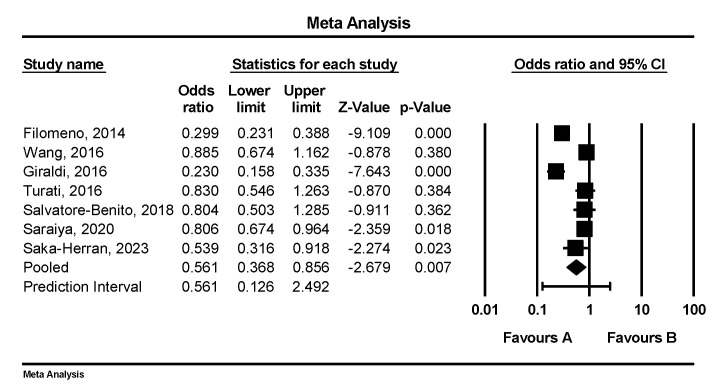
Adherence to the Mediterranean diet and HNC risk (*n* = 7). Q = 74.652, df = 6, *p* < 0.001 [29,30,31,32,33,34,35].

The initial analysis of eight studies (*n* = 8) demonstrated that a three-unit increase in the MD adherence score was significantly associated with a decreased risk for head and neck cancer (pooled OR = 0.565, 95% CI: 0.388–0.822, *p* = 0.003, I^2^ = 91%) (Supplementary Figure S1). After the exclusion of Samoli et al. [27], the pooled OR was similar: 0.561 (95% CI: 0.368–0.856, *p* = 0.007, I^2^ = 92%) (Figure 2). Moreover, a study by Bosetti et al. [37,38,39], which was not included in the meta-analysis as it was published more than 15 years ago, found that high adherence to the MD significantly reduced the risk of upper aerodigestive tract cancers. Specifically, individuals with six or more MD characteristics had a 60% reduced risk of oral and pharyngeal cancer (OR = 0.40, 95% CI: 0.29–0.54) and a 77% reduced risk of laryngeal cancer (OR = 0.23, 95% CI: 0.14–0.36) compared to those with fewer than three characteristics.

### 3.6. Head and Neck Cancer Survival

One study by Saraiya et al. [28] investigated the association between pre-diagnosis adherence to the Mediterranean diet, as evaluated by the Mediterranean Diet Score (MDS), and mortality among 1184 individuals diagnosed with head and neck cancer. The 5-year survival rates for all-cause mortality and HNC-specific mortality for the highest versus lowest MDS quintiles were [HR (95% CI): 0.51 (0.33, 0.80); *p* = 0.014] and [HR (95% CI): 0.43 (0.22, 0.85); *p* = 0.004], respectively. Specifically, a one-unit increase in MDS adherence was associated with a 15% reduction in the 5-year hazard ratio (HR) for HNC-specific death for tumors located in the oral cavity [HR (95% CI): 0.85 (0.75, 0.96)] (Table 1). Additionally, a study by Crosignani et al. [36] demonstrated that male laryngeal cancer patients who adhered to a Mediterranean diet experienced a 36% increase in 10-year survival, highlighting the potential benefits of MD interventions in improving outcomes.

### 3.7. Individual Dietary Component/Food Group Analysis

**Figure 3:**
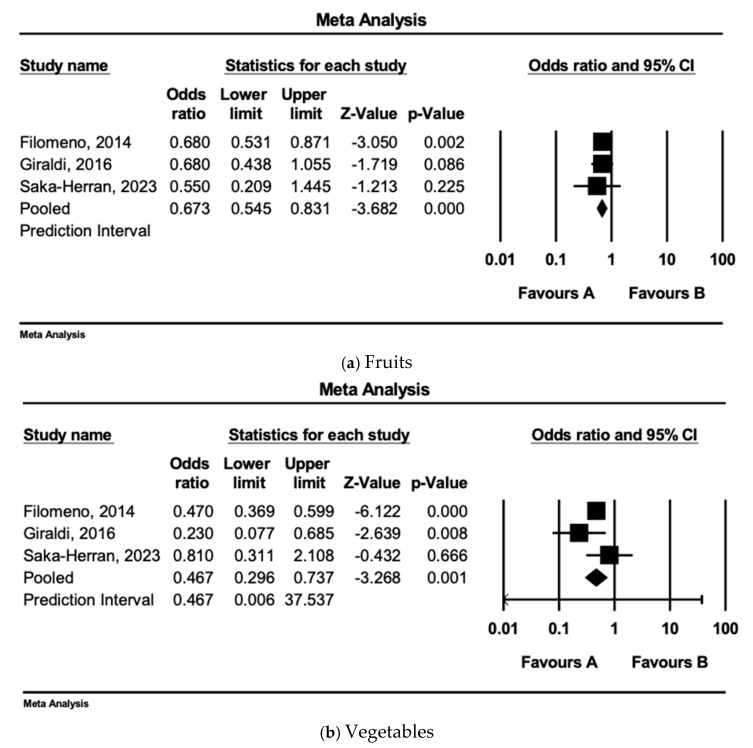

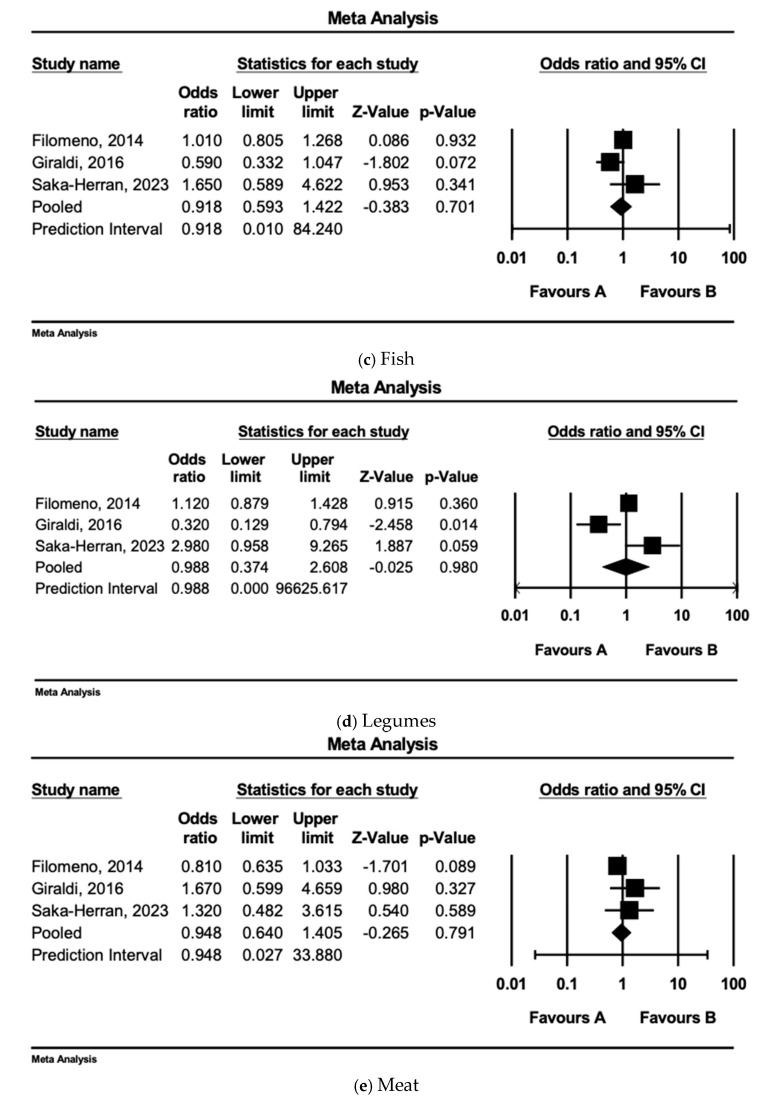
(**a**) Adherence to dietary components (fruits) and HNC risk (*n* = 3). Q = 0.176, df = 2, Ι^2^ set to 0. (**b**) Adherence to dietary components (vegetables) and HNC risk (*n* = 3). Q = 2.89, df = 2 and *p* = 0.236, Ι^2^ = 31%. (**c**) Adherence to dietary components (fish) and HNC risk (*n* = 3). Q = 4.017, df = 2 and *p* = 0.134, Ι^2^ = 50%. (**d**) Adherence to dietary components (legumes) and HNC risk (*n* = 3). Q = 10.047, df = 2 and *p* = 0.007, Ι^2^ = 50%. (**e**) Adherence to dietary components (meat) and HNC risk (*n* = 3). Q = 2.536, df = 2 and *p* = 0.281, Ι^2^ = 21% [32,34,35].

Three studies reported results from the analysis of specific food groups, including fruits, vegetables, fish, legumes, and meat [32,34,35]. A subgroup analysis of these studies indicated that high fruit and vegetable intake was significantly associated with a reduced risk of head and neck cancer [HR (95% CI): 0.67 (0.55, 0.83) and HR (95% CI): 0.47 (0.30, 0.74 respectively)] (Figure 3a,b). In contrast, a high intake of legumes [HR (95% CI): 0.99 (0.37, 2.6)] and fish [HR (95% CI): 0.92 (0.59, 1.42)] and low meat consumption [HR (95% CI): 0.95 (0.64, 1.4)] were not associated with a statistically significant decreased risk of head and neck cancer (Figure 3c–e).

## 4. Discussion

The results of this meta-analysis support a significant association between higher adherence to the Mediterranean diet (MD) and a lower risk of head and neck cancer (HNC). Specifically, a three-unit increase in MD adherence scores was associated with a more than 40% lower HNC risk (pooled OR = 0.561, 95% CI: 0.368–0.856, *p* = 0.007, I^2^ = 92%). Additionally, analysis of individual MD components revealed that high fruit and vegetable intake were both associated with decreased HNC risk, while the intake of legumes, fish, and meat did not show a significant association.

For this meta-analysis, only studies published within the last 15 years were included to account for significant changes in smoking trends [20], dietary habits [27], and the prevalence of obesity [22], all of which could influence the relationship between dietary adherence and cancer risk. These shifts reflect global public health patterns, such as declining smoking rates, the increasing prevalence of ultra-processed foods, and rising obesity rates, which could impact recent study findings. In contrast, the systematic review was not restricted by publication date, which allowed the inclusion of additional studies. Notably, a study by Crosignani et al. [36] found that individuals whose diet closely adhered to the Mediterranean diet had a 60% reduced risk of oral and pharyngeal cancer and a 77% reduced risk of laryngeal cancer compared to those with lower adherence. These previous findings align with the results of our contemporaneous meta-analysis, further supporting the protective role of the MD against head and neck cancers.

Our findings are in agreement with existing literature supporting the protective role of the MD against various cancer types and support the potential of dietary interventions as part of comprehensive strategies for HNC prevention. Previous epidemiological studies have demonstrated the benefits of the MD in reducing the risk of other cancers, such as breast, colorectal, and prostate cancer. For instance, in breast cancer, the PREDIMED trial found a 68% risk reduction in the group adhering to the MD with extra virgin olive oil (EVOO) [46]. Other studies have also shown a reduced breast cancer risk in both premenopausal and postmenopausal women adhering to the MD [47,48]. Regarding lung cancer, a meta-analysis revealed a 9% risk reduction for every three-point increase in MD score [49], and another study showed a 16% reduction in lung cancer risk with long-term adherence [50]. For gastric cancer, a meta-analysis reported a 43% risk reduction [51], with additional studies also supporting this protective effect [52,53]. Decreased pancreatic cancer risk (18%) was associated with higher MD adherence in a recent meta-analysis [54], and a meta-analysis confirmed an 18% reduction in colorectal cancer with greater MD adherence [53]. While findings for prostate cancer have been mixed, recent data from the North Carolina-Louisiana Prostate Cancer Project indicated an inverse association between MD adherence and high-risk prostate cancer [55].

Other current literature on dietary habits not specific to the Mediterranean diet and HNC risk also supports a protective role of healthy dietary patterns. Notably, lower HNC risk was associated with greater consumption of whole grains (HR = 0.78; 95% CI: 0.64–0.94/oz per day) and whole fruits (HR = 0.90; 95% CI: 0.82–0.98/cup per day), as well as overall healthy eating (HR = 0.87; 95% CI: 0.78–0.98/10 points) [56]. Conversely, a higher consumption of ultra-processed foods (UPF) has been associated with an increased risk of head and neck cancer (HNC) in the European Prospective Investigation into Cancer and Nutrition (EPIC) cohort [57]. Moreover, in a recent prospective study of 476 newly diagnosed Head and Neck Squamous Cell Carcinoma patients, the results demonstrated that prognosis might vary depending on the fat types consumed before cancer treatment, with higher unsaturated fat intake being associated with a reduced all-cause and disease-specific mortality risk. High intakes of ω-3 PUFAs were also significantly associated with reduced all-cause mortality in these patients [17]. Additionally, in a prospective study, higher adherence to the Mediterranean diet (aMED score) showed a protective effect against head and neck cancer, with a 20% risk reduction in men and a 58% risk reduction in women when comparing the highest adherence levels to the lowest [58].

Additionally, evidence suggests that the MD may benefit survival outcomes in HNC patients. Saraiya et al. [28] investigated the association between pre-diagnosis adherence to the MD and mortality among individuals diagnosed with HNC. They found significantly lower 5-year all-cause mortality [HR (95% CI): 0.51 (0.33, 0.80); *p* = 0.014] and HNC-specific mortality [HR (95% CI): 0.43 (0.22, 0.85); *p* = 0.004] in the highest versus lowest MDS quintiles. Similarly, Crosignani et al. [36] demonstrated a 36% increase in 10-year survival among male laryngeal cancer patients adhering to the MD, underscoring the potential of MD interventions in improving outcomes.

The Mediterranean diet (MD) emphasizes plant-based foods and healthy fats and limits red and processed meats, thereby reducing oxidative stress and inflammation, which are key factors in carcinogenesis. Antioxidants and anti-inflammatory nutrients from legumes, fruits, vegetables, nuts, fish, and extra virgin olive oil help prevent cancer by counteracting DNA damage and inhibiting cancer cell growth and metastasis through polyphenols and omega-3 fatty acids [59,60,61,62,63,64,65]. Reducing meat intake further protects by limiting exposure to harmful compounds from high-temperature cooking and reducing animal fat, which are linked to a higher cancer risk [66]. Beyond its anti-inflammatory effects, the MD also lowers lipid levels, modulates cancer-related hormones and growth factors, and supports a beneficial gut microbiota that aids cancer prevention [67,68,69,70]. By integrating diet with physical activity and other lifestyle factors, the MD plays a significant role in reducing cancer risk [71,72].

To the best of our knowledge, this is the first meta-analysis investigating the association between Mediterranean diet adherence and head and neck cancer risk. A key strength of our study is its comprehensive approach, which included data from multiple countries and utilized validated diet assessment scores (e.g., MDS, aMED, MEDAS) to ensure consistency, allowing for a robust evaluation of the association between MD adherence and HNC risk. Additionally, subgroup analyses of dietary components provided insights into specific elements of the MD that may drive the observed protective effect, highlighting potential areas for focused dietary recommendations.

However, certain limitations should be considered. The high heterogeneity observed among studies suggests variability in study design, populations, and dietary assessment methods. Additionally, all included studies were observational case–control studies, which are inherently susceptible to recall bias and reverse causality. Although we used the Newcastle–Ottawa Scale to assess methodological quality, residual confounding from unmeasured factors, such as physical activity and other lifestyle habits, cannot be ruled out. Furthermore, although different MD adherence scores measure similar variables, they may still introduce subtle differences in how adherence is assessed across studies. Finally, although covariates were accounted for in all the studies included in the meta-analysis (Supplementary Table S2), variability in the specific covariates considered across studies introduces potential bias. Not all studies accounted for the same set of covariates, and some may have omitted important ones.

In conclusion, this meta-analysis supports a significant inverse association between MD adherence and HNC risk, suggesting that the MD may serve as a promising dietary approach for reducing HNC incidence. Promoting the MD, which is characterized by high intakes of fruits, vegetables, legumes, and healthy fats, may be a valuable strategy in comprehensive cancer prevention efforts. Public health initiatives to promote MD adherence could include education on plant-based diets, increasing accessibility to MD foods (e.g., fruits, vegetables, legumes), and developing culturally appropriate dietary guidelines that emphasize MD principles.

Future studies should consider employing prospective cohort designs and randomized controlled trials to minimize recall bias and provide stronger evidence of causality. Additionally, randomized controlled trials focusing on dietary interventions in high-risk populations could clarify the effectiveness of Mediterranean diet adherence in reducing HNC incidence. Furthermore, research into the molecular mechanisms by which the Mediterranean diet and its components exert anticancer effects in HNC may lead to more targeted dietary recommendations and interventions.

## Figures and Tables

**Table 1:** Study characteristics.

Author, Year	Country	Type of Study	Cases/	Age (Years ± SD or IQR)		Sex (Males/Females)		Year of Diagnosis	Mediterranean Diet Adherence Score	Type of Cancer	Outcome
			Controls (n)								
				Cases	Controls	Cases	Controls				
Crosignani et al., 1996 [36]	Italy	Case–control, Population-based	220/0	59 (32–75)	-	220/0	No control	Registry	Tertiles of intake of various food groups	Laryngeal cancer	All-cause mortality
Bosetti et al., 2003 [37,38,39]	Italy	Case–control	598/1492	58 (22–77)	58 (20–78)	512/87	1008/484	1992–1998	An MD score was defined on the basis of eight characteristics of the traditional MD. Adherence was stratified as <3, 3, 4, 5, and ≥6	Cancers of the oral cavity and pharynx	OCP cancer risk
	Italy and Switzerland	Case–control	457/1087	61 (30–79)	61 (31–79)	478/49	1052/245	1992–2000		Cancer of the larynx	Laryngeal cancer risk
Samoli et al., 2010 [27]	Greece	Case–control	239/194	61.3 ± 0.8	60.6 ± 1.0	192/47	143/51	2002–2005	MDS (two- or three-unit increase)	Cancers of the oral cavity, pharynx (excluding nasopharynx), larynx and esophagus	Upper aerodigestive tract cancer risk
Filomeno et al., 2014 [35]	Italy and Switzerland	Case–control	768/2078	58 (22–79)	59 (19–79)	593/175	1368/710	1997–2009	MDS (≤2 vs. ≥6	Cancers of the oral cavity and pharynx (OCP)	OCP cancer risk
									MDP (≤57.9 vs. ≥66.2)		
									MAI (≤0.92 vs. ≥2.1)		
Giraldi et al., 2016 [34]	Italy	Case–control	500/433	63.1 ± NA	58.8 ± NA	402/98	254/179	2002–2014	MDS (Continuous)	Cancers of the oral cavity, oropharynx, hypopharynx, and larynx	Head and neck cancer risk
Turati et al., 2016 [30]	Italy	Case–control	198/594	52 (18–76)	52 (19–76)	157/41	471/123	1992–2008	MDS (≤4 vs. 5 and ≤4 vs. ≥6)	Nasopharyngeal cancer	NPC risk
Wang et al., 2016 [31]	China	Case–control	600/600	47.4 ± 9.0	47.4 ± 9.0	448/152	448/152	2009–2011	aMed (≤2 vs. ≥6)	Nasopharyngeal cancer	NPC risk
Salvatore-Benito et al., 2018 [29]	Spain	Case–control	68/100	64.9 ± 9.7	59.7 ± 17.9	58/10	44/56	2018	MEDAS [≤7 (poor adherence) vs. 8–9 (moderate adherence) vs. ≥10 (good adherence)]	Head and neck cancers	HNC risk
Saraiya et al., 2020 [33]	USA	Case–control	1170/1303	No mean/median reported	No mean/median reported	899/271	904/399	2002–2006	MDS, 1 SD unit change (SD = 1.7)	Oral cavity, pharynx, and larynx	HNC risk
									MDS-HNC, 1 SD unit change (SD = 2.2)		
Saka-Herran et al., 2023 [32]	Spain	Case–control	101/101	66 ± 10.2	65.8 ± 9.9	69/32	69/32	2018–2022	MD questionnaire that was adapted from the Spanish Society of Atherosclerosis [unhealthy (≤4) vs. healthy (10–14) vs. regular (5–9)]	Head and Neck	HNC risk
Saraiya et al., 2024 [28]	USA	Case–control, Population-based, cases linked to the National Death Index, 1:1	1184/0	No mean/median reported	-	912/272	No control	2002–2006	MDS highest versus lowest quintile	Oral cavity, pharynx, and larynx	All-cause and HNC-specific mortality

## Data Availability

No new data were created or analyzed in this study. Data sharing is not applicable to this article.

## Supplementary Materials

The following supporting information can be downloaded at https://www.mdpi.com/article/10.3390/nu17020287/s1: Figure S1: Adherence to the Mediterranean Diet and HNC risk (*n* = 8) [Sensitivity analysis]; Table S1: Methodological assessment of the included studies according to the Newcastle-Ottawa scale; Table S2: Study inclusion criteria and covariates; Table S3: Harmonization methods for the different MD scores.
