# Supplementary material for: Adherence to the Mediterranean Diet and the Risk of Head and Neck Cancer: A Systematic Review and Meta-Analysis of Case–Control Studies
Source: Nutrients. 2025 Jan 14;17(2):287. doi: 10.3390/nu17020287 (PMC11767401; doi:10.3390/nu17020287)
Supplement: Supplementary file 1 [file nutrients-17-00287-s001.zip › nutrients-3380312-supplementary.pdf]

Supplementary Materials

Table S1. Methodological assessment of the included studies according to the Newcastle- Ottawa Scale.

Newcastle-Ottawa Assessment Scale									
	Selection					Outcome			
Author; Date	Definition of Cases	Representativeness of Cases	Selection of Controls	Definition of Controls	Comparability	Ascertainment of Exposure	Same Methods of Ascertainment for Cases and Controls	Non-Response rate	Total
Crosignani et al, 1996 [36]	·	·		·	··	·	·		7
Bosetti et al, 2003 [37–39]	·	·		·	··	·	·		7
Samoli E. et al; 2010 [27]	·	·		·	··		·		7
Filomeno M. et al; 2014 [35]	·	·		·	··	·	·		7
Wang C. et al; 2016 [34]	·	·		·	··		·		6
Giraldi, L. et al; 2017 [30]	·			·	··	·	·	·	7
Turati F. et al; 2017 [31]	·	·		·	··		·	·	7
Salvatore Benito A. et al; 2019 [29]	·	·	·	·	··		·		7
Saraiya V. et al; 2020 [33]	·	·		·	·	·			5
Saka-Herrán C. et al; 2023 [32]	·			·	··	·	·		6
Saraiya, V. et al; 2024 [28]	·	·	·	·	··		·		7

Table S2. Study inclusion criteria and covariates.

Author, Year	Inclusion Criteria	Covariates Accounted for
Crosignani et al., 1996 [36]	Cases were resident in the area of the Lombardy Cancer Registry and diagnosed with laryngeal cancer.	Age at diagnosis, clinical stage, and occurrence of new primaries
Bosetti et al, 2003 [37-39]	Cases had histologically confirmed cancer of the oral cavity and pharynx diagnosed no longer than 1 year before the interview and with no previous diagnoses of cancer at any site. Controls were patients with no history of cancer who were admitted to major hospitals in the same catchment areas as cases and who had acute, non-neoplastic conditions unrelated to smoking, alcohol drinking, and long-term modifications of diet.	Age, sex, study center, years of education, tobacco consumption, body mass index, and total energy intake
Samoli et al,2010 [27]	Cases: pathologically confirmed cancer of the oral cavity or pharynx (excluding nasopharynx), cancer of the larynx, and cancer of the esophagus, admitted in four major hospitals<br>Controls: Subjects with a recently diagnosed non-malignant disease in the same hospital unrelated to alcohol, tobacco or dietary practices	Age, sex, height, BMI, educational level, smoking status, pack years, and energy intake
Filomeno et al, 2014 [35]	Cases: incident, histologically confirmed squamous cell cancers of the oral cavity and pharyngeal cancer (excluding cancers of the lip, salivary glands, and nasopharynx), admitted to major teaching or general hospitals in the areas under investigation.<br>Controls: subjects with no previous history of cancer, admitted to the same hospitals as cases for acute, non-neoplastic conditions, unrelated to tobacco smoking, alcohol drinking, or long-term dietary modifications.	Age, sex, study center, year of interview, education, smoking, BMI, total energy intake
Giraldi et al, 2016 [34]	Cases: patients with tumors of the oral cavity, oropharynx, hypopharynx, and larynx who were admitted to a university hospital.<br>Controls: Patients admitted to the same hospital as the cases, in the same period, for a condition unrelated to cancer.	Age, sex, tobacco smoking, alcohol drinking, and total energy intake
Turati et al, 2016 [30]	Cases: patients admitted to major teaching and general hospitals in the study areas, with an incident histologically confirmed diagnosis of nasopharyngeal cancer (NPC), and without previous history of cancer at other sites.<br>Controls: patients admitted to the same hospitals as cases for a wide spectrum of acute, non-neoplastic diseases, unrelated to known risk factors for NPC, including tobacco smoking and alcohol drinking, as well as long-term dietary modifications.	Age, sex, and place of residence (model 1), and further for education, tobacco smoking, and total energy intake
Wang et al, 2016 [31]	Cases: Inpatients at the Sun Yat-sen University Cancer Center with histological diagnoses of NPC up to 3 months before interviews	Age, BMI, occupation, marital status, educational level, household income,

	Controls: Inpatients hospitalized in the Sun Yat-sen University Ophthalmic Center within 1 week	smoking, drinking, toxic substances, multivitamin, chronic rhinitis history, physical activity, daily energy intake, preserved vegetables and animal food
Salvatore-Benito et al, 2018 [29]	Cases: patients over 18 years of age diagnosed with HNC in the first 6 months of 2018<br>Controls: Healthy individuals without HNC.	Age, gender, smoking, alcohol abuse,income,education level
Saraiya et al. 2020 [33]	Cases: age ranged between 20-80 years at the time of diagnosis resided within a 46-country region in central and eastern North Carolina and was diagnosed with primary invasive squamous cell carcinoma of the oral cavity, pharynx, or larynx between Jan 1, 2002, to Feb 28, 2006<br>Controls: resided in the same counties that were frequency-matched with cases.	Age, race, sex, BMI, history of loose teeth, educational attainment, smoking, alcohol, energy intake
Saka-Herran et al, 2023 [32]	Cases: Older than 18 years old, able to respond to a questionnaire, and able to give their informed consent<br>Controls: The inclusion criteria were that the participants be older than 18 years old, able to respond to a questionnaire, and able to give their informed consent.	Education, monthly income, smoking status, alcohol consumption, physical activity, and comorbidities
Saraiya et al, 2024 [28]	Cases: age ranged between 20-80 years at the time of diagnosis and resided within a 46-country region in central and eastern North Carolina and was diagnosed with primary invasive squamous cell carcinoma of the oval cavity, pharynx, or larynx between Jan 1, 2002, to Feb 28, 2006	Age, race, sex, BMI, education, smoking, alcohol intake, stage, quartile of energy intake

Table S3. Harmonization methods for the different MD scores.

Author (year)	NOTES
Amoli et al, 2010 [27]	3-unit increase in MDS score
Filomeno et al, 2014 [35]	Used MDS (≤2 vs. ≥6) which we approximated as a 4-unit increase and converted it to a 3-unit increase.
Giraldi et al, 2016 [34]	Presented a continuous MDS with a 1-unit increase, which was converted to a 3-unit increase.
Turati et al, 2016 [30]	Used MDS (≤4 vs. 6), which was converted to a 3-unit increase.
Wang et al, 2016 [31]	Used aMED (≤2 vs. ≥6), which we approximated as a 4-unit increase, then converted to a 3-unit increase. A 3-unit increase in aMED was considered equivalent to a 3-unit increase in MDS.
Salvatore-Benito et al, 2018 [29]	Employed MEDAS and presented results for a continuous 1-unit increase, which was converted to a 3-unit increase and assumed to correspond to a 3-unit increase in MDS.
Saraiya et al, 2020 [33]	Used continuous MDS based on an SD change (1.7 units), which was converted to a 3-unit increase.
Saka-Herran et al, 2023 [32]	Used a Mediterranean diet adapted by the Spanish Society of Atherosclerosis (≤4 vs. ≥10). It was converted to a 3-unit increase, assuming that a 3-unit increase in the score used is equivalent to a 3-unit increase in the MDS.

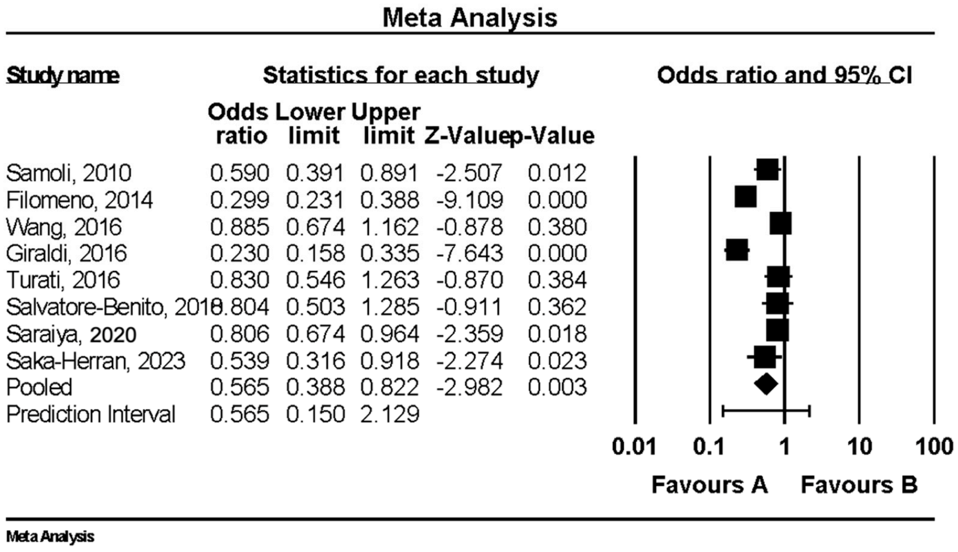

$Q = 74.659$, $df = 7$, $p < 0.001$.

Figure S1. Adherence to the Mediterranean Diet and HNC risk ($n = 8$) [Sensitivity analysis].
